# Editorial: Stress, mood, and fatigue: Tackling “invisible” obstacles in stroke rehabilitation and recovery

**DOI:** 10.3389/fneur.2022.1121667

**Published:** 2023-01-17

**Authors:** Prajwal Gyawali, Dana Wong, Brenton Hordacre, Lin Kooi Ong, Coralie English

**Affiliations:** ^1^School of Health and Medical Sciences and Centre for Health Research, University of Southern Queensland, Toowoomba, QLD, Australia; ^2^School of Psychology and Public Health, La Trobe University, Melbourne, VIC, Australia; ^3^Innovation, Implementation and Clinical Translation in Health (IIMPACT in Health), Allied Health and Human Performance, University of South Australia, Adelaide, SA, Australia; ^4^School of Biomedical Sciences and Pharmacy, The University of Newcastle, Newcastle, NSW, Australia; ^5^School of Pharmacy, Monash University Malaysia, Selangor, Malaysia; ^6^Heart and Stroke Research Program, Hunter Medical Research Institute, Newcastle, NSW, Australia; ^7^School of Health Sciences, The University of Newcastle, Newcastle, NSW, Australia

**Keywords:** stress, depression, fatigue, resiliency, rehabilitation

More than one-half of stroke survivors experience ongoing psychological difficulties such as stress, fatigue, anxiety, and depression (1, 2). These ongoing issues create barriers to stroke recovery and rehabilitation. Post-stroke fatigue and depression are associated with increased mortality (3, 4), decreased quality-of-life (5) and poorer functional outcome (6, 7). Similarly, perceived stress is associated with long-term poorer functional outcome (8, 9). Despite being important determinants of stroke outcome and recovery, stress and fatigue are often not specifically addressed as part of stroke rehabilitation programmes. There are limited evidence-based programmes that have been designed to empower and enable stroke survivors to manage these conditions. Likewise, post-stroke depression remains largely underdiagnosed and untreated (10). This may be due to the fact that unlike physical disabilities, these psychological difficulties are “invisible.” This Research Topic was dedicated to this healthcare gap in stroke rehabilitation and the articles published in this issue showcases the clear need to tackle these invisible obstacles.

There is the lack of consensus regarding the nature and type of support that people with stroke might need to manage their fatigue. This issue contains a qualitative study by Bicknell et al. who describe the experience of post-stroke fatigue during outpatient rehabilitation from the perspective of people with stroke and their carers. Six major themes were identified. The findings suggested that most stroke survivors had to find their own solutions to manage their post-stroke fatigue, and support from care-givers, can lead to both positive and negative experiences. The authors concluded that post-stroke fatigue should be routinely screened, and appropriate support and education should be provided during rehabilitation.

The paper by Kliem et al., included in this issue, focused on characterizing the inter-relationships between fatigue, sleep, cognitive functioning and mood. They found that poorer cognition and lower mood at 3 months post-ischemic stroke predicted worse fatigue and daytime sleep at 12 months, controlling for stroke severity, age, sex, and difficulties sleeping at night. As highlighted by the authors, this suggests that interventions addressing both cognition and mood, such as Cognitive Behavior Therapy, may be a promising approach to treating fatigue and sleep difficulties (11, 12).

Perceptual disorders are not uncommon after stroke (13). In this Research Topic, Chen et al. investigated whether perceived limb heaviness can affect engagement in rehabilitation after stroke. By retrospectively reviewing 108 participants data, they found 37% reported limb heaviness and this, along with strength, appeared to influence engagement in rehabilitation. Solutions targeting altered body perceptions might have a role in promoting better stroke recovery.

Repetitive transcranial magnetic stimulation (rTMS) is a treatment for depression that has shown promise, however relatively few studies have explored its use in post-stroke depression. It is thought that stimulation of the left dorsolateral prefrontal cortex might initiate early stages of synaptic plasticity, supporting improvements in mood. However, it is unclear whether damage to this brain region due to stroke influences efficacy of this treatment. In a case study published in the current issue, Hordacre et al. applied rTMS as a treatment for post-stroke depression in a patient with ischemic damage to the left middle frontal gyrus. Comprising an acute, daily treatment schedule, followed by weekly maintenance, depression scores were meaningfully reduced, with benefits persisting well beyond completion of the treatment. rTMS appears worthy of further investigation as a treatment for post-stroke depression.

A growing body of evidence has indicated that higher measures of resilience are associated with the lower levels of perceived stress and recovery after stroke (8). However, the study of Norvang et al., included in this issue, did not find an association of early measurement of resilience obtained within 2 weeks after stroke with activities of daily living at 3 months post-stroke. The authors suggested that the impact of resilience might not apply to physical adversity as compared to psychosocial adversity.

Preliminary evidence suggests that traditional Chinese medicines may also have a role in reducing post-stroke depression. Although not clear, preliminary work suggests acupuncture might evoke similar changes in neurotransmitter activity and brain connectivity as pharmacological management of depression (14, 15). In this issue, Luo et al. proposed a protocol for a randomized controlled trial to evaluate efficacy and safety of optimized acupuncture and moxibustion as a treatment for post-stroke depression. The trial will aim to recruit 134 patients, randomized 1:1 to intervention or control. Treatments will be delivered five times per week for 4 weeks.

Racial disparities exist in stroke treatment and care (16). In this issue, Love et al. provided a protocol that aim to understand if resilience could buffer the effect of racism and psychological stress on quality of life among black stroke survivors in USA.

Taken together, the articles in this special edition provide interesting insights that build our understanding of the invisible obstacles to stroke recovery and rehabilitation including stress, depression, mood, and fatigue. Several articles suggested potential interventions and trial protocols to mitigate these invisible obstacles, including Cognitive Behavior Therapy, resilience building programs, acupuncture and repetitive transcranial magnetic stimulation. Future studies are needed to better understand the prevalence, pathophysiology, inter-relationships, mechanisms, impacts and trajectory of these issues and more rigorous clinical trials are warranted to investigate the feasibility and efficacy of potential therapeutic interventions. We hope that this Research Topic provides a springboard to encourage more work in this important area.

## Author contributions

All authors listed have made a substantial, direct, and intellectual contribution to the work and approved it for publication.

## References

[1] Zhan J Zhang P Wen H Wang Y Yan X Zhan L. Global prevalence estimates of poststroke fatigue: a systematic review and meta-analysis. Int J Stroke. (2022) 2022:17474930221138701. 10.1177/17474930221138701 36314998

[2] Guo J Wang J Sun W Liu X. The advances of post-stroke depression: 2021 update. J Neurol. (2021) 2021:1–14. 10.1007/s00415-021-10597-4 34052887

[3] Glader E-L Stegmayr B Asplund K. Poststroke fatigue: a 2-year follow-up study of stroke patients in Sweden. Stroke. (2002) 33:1327–33. 10.1161/01.STR.0000014248.28711.D6 11988611

[4] Cai W Mueller C Li Y-J Shen W-D Stewart R. Post stroke depression and risk of stroke recurrence and mortality: a systematic review and meta-analysis. Ageing Res Rev. (2019) 50:102–9. 10.1016/j.arr.2019.01.013 30711712

[5] Naess H Lunde L Brogger J. The effects of fatigue, pain, and depression on quality of life in ischemic stroke patients: the Bergen Stroke Study. Vasc Health Risk Manag. (2012) 8:407. 10.2147/VHRM.S32780 22910531 PMC3402053

[6] Christensen D Johnsen SP Watt T Harder I Kirkevold M Andersen G. Dimensions of post-stroke fatigue: a two-year follow-up study. Cerebrovasc Dis. (2008) 26:134–41. 10.1159/000139660 18560216

[7] Schöttke H Gerke L Düsing R Möllmann A. Post-stroke depression and functional impairments–a 3-year prospective study. Compr Psychiatry. (2020) 99:152171. 10.1016/j.comppsych.2020.152171 32179262

[8] Gyawali P Chow WZ Hinwood M Kluge M English C Ong LK. Opposing associations of stress and resilience with functional outcomes in stroke survivors in the chronic phase of stroke: a cross-sectional study. Front Neurol. (2020) 11:230. 10.3389/fneur.2020.00230 32390923 PMC7188983

[9] Ostwald SK Bernal MP Cron SG Godwin KM. Stress experienced by stroke survivors and spousal caregivers during the first year after discharge from inpatient rehabilitation. Top Stroke Rehabil. (2009) 16:93–104. 10.1310/tsr1602-93 19581196 PMC2748854

[10] Medeiros GC Roy D Kontos N Beach SR. Post-stroke depression: a 2020 updated review. Gen Hosp Psychiatry. (2020) 66:70–80. 10.1016/j.genhosppsych.2020.06.011 32717644

[11] Nguyen S Wong D McKay A Rajaratnam SM Spitz G Williams G. Cognitive behavioural therapy for post-stroke fatigue and sleep disturbance: a pilot randomised controlled trial with blind assessment. Neuropsychol Rehabil. (2019) 29:723–38. 10.1080/09602011.2017.1326945 28521579

[12] Ymer L McKay A Wong D Frencham K Grima N Tran J. Cognitive behavioural therapy versus health education for sleep disturbance and fatigue after acquired brain injury: a pilot randomised trial. Ann Phys Rehabil Med. (2021) 64:101560. 10.1016/j.rehab.2021.101560 34311119

[13] Serrada I Hordacre B Hillier S. Recovery of body awareness after stroke: an observational study. Front Neurol. (2021) 12:745964. 10.3389/fneur.2021.745964 34912283 PMC8666978

[14] Leung MCP Yip KK Ho YS Siu FKW Li WC Garner B. Mechanisms underlying the effect of acupuncture on cognitive improvement: a systematic review of animal studies. J Neuroimmune Pharmacol. (2014) 9:492–507. 10.1007/s11481-014-9550-4 24903518

[15] Liu P Qin W Zhang Y Tian J Bai L Zhou G. Combining spatial and temporal information to explore function-guide action of acupuncture using fMRI. J Magn Reson Imaging. (2009) 30:41–6. 10.1002/jmri.21805 19557845

[16] Cruz-Flores S Rabinstein A Biller J Elkind MS Griffith P Gorelick PB. Racial-ethnic disparities in stroke care: the American experience: a statement for healthcare professionals from the American Heart Association/American Stroke Association. Stroke. (2011) 42:2091–116. 10.1161/STR.0b013e3182213e24 21617147

